# Sex-dependent relationship of C-reactive protein levels with HDL-cholesterol and HDL-phospholipid concentrations in children

**DOI:** 10.1038/s41598-022-07271-8

**Published:** 2022-02-25

**Authors:** Claudia Vales-Villamarín, Olaya de Dios, Iris Pérez-Nadador, Teresa Gavela-Pérez, Leandro Soriano-Guillén, Carmen Garcés

**Affiliations:** 1 Lipid Research Laboratory, IIS-Fundación Jiménez Díaz, 28040 Madrid, Spain; 2 Department of Pediatrics, IIS-Fundación Jiménez Díaz, 28040 Madrid, Spain

**Keywords:** Predictive markers, Lipoproteins, Risk factors

## Abstract

Obesity has been consistently associated with inflammation but the influence of HDL on this association remains under study. Our study analyzes the influence of obesity-related parameters in the relationship of high-sensitivity C-reactive protein (hs-CRP) with HDL-cholesterol and HDL-phospholipid in male and female adolescents. The study sample population comprised 350 males and 401 females aged 12 to 16 years. Information regarding anthropometric parameters, HDL-cholesterol, HDL-phospholipid, adiponectin, leptin, insulin, and hs-CRP concentrations was available. hs-CRP levels were inversely related to HDL-cholesterol and HDL-phospholipid in males but not in females, and were positively related to leptin concentrations in both sexes but were not related to adiponectin levels. In regression analyses, HDL-phospholipid and leptin appeared significantly associated to hs-CRP in males in a model explaining 14.3% of hs-CRP variation. In females, only leptin appeared related to hs-CRP concentrations. After adjusting by leptin and adiponectin, males in the highest hs-CRP tertile showed significantly lower levels of HDL-cholesterol and HDL-phospholipid than those in tertiles 1 and 2, while no significant differences in HDL-cholesterol and HDL-phospholipid concentrations by hs-CRP tertile were observed in females. In summary, high hs-CRP levels were associated with lower plasma HDL-cholesterol and HDL-phospholipid concentrations in male adolescents irrespective of adipokines, while in females, HDL-related parameters are not associated with hs-CRP concentrations*.*

## Introduction

Pathological processes related to the development of arteriosclerosis begin in early life^1^. The systemic low-grade inflammatory state related to obesity in children is associated with the early stages of atherogenesis^2^. C-reactive protein (CRP), the best studied marker of subclinical inflammation, has been shown to be a good predictor of cardiovascular risk when measured by high-sensitivity assays (hs-CRP)^3^ and has been linked to the presence of the metabolic alterations leading to cardiovascular diseases^4^. Serum CRP concentrations have been consistently associated with obesity as well as with obesity-related alterarions in adults^5–7^ and children^7–10^.

On the other hand, the low-grade inflammation associated with obesity has been linked to the dysfunction of HDL^11^. The relationship of HDL-cholesterol levels with the development of atherosclerosis and the risk of coronary heart disease (CHD) has been long established^12,13^. HDL can protect against atherosclerosis and CHD by means of its role in the reverse cholesterol transport but also in the LDL modification process^14^ affecting the oxidative stress related to the inflammatory response. An important role in this association between oxidative stress and the inflammatory response has been attributed to CRP^15^. Previous studies of our group have shown an association of higher hs-CRP levels with lower HDL-cholesterol in prepubertal children and 12- to 16-year-old males but not in 12- to 16-year-old females^16^. It is accepted that adipose tissue is not just a simple reservoir of energy but also an organ actively involved in metabolic control by secreting adipocytokines which play important biological roles^17^. Adiponectin appears as one of the adipose tissue-derivate cytokines acting as an anti-inflammatory factor^18,19^, by means to its positive effects on glucose and lipid metabolism^20^ and insulin sensitivity^21^, that could contribute to the relationship between CRP and HDL-cholesterol and HDL-phospholipid. We have previously identified adiponectin as an important determinant of HDL-cholesterol and HDL-phospholipid concentrations in pubertal children, particularly in females^22^. Leptin is another adipose tissue related cytokine that plays an important role in the development of obesity and obesity-related complications such as altered lipid profile^23^. Leptin has been postulated as one of the mediators of inflammation involved in inflammatory disorders^19,24^. A positive correlation between CRP and leptin levels has been reported^25^.

In our study, we aimed to investigate the independent relationship of plasma hs-CRP levels with HDL-cholesterol and HDL-phospholipid concentrations in a population-based sample of males and females from 12 to 16 years of age.

## Results

The study included 350 males and 401 females. The characteristics of the study participants are summarized in Table 1.

**Table 1:** Characteristic (mean ± SD) of the study participants.

	Males (n = 350)	Females (n = 401)	p-value
Age (years)	14.4 ± 1.2	14.4 ± 1.1	NS
BMI (kg/m^2^)	22.1 ± 4.0	21.9 ± 3.5	NS
BMI z-score	0.21 ± 1.08	0.20 ± 1.01	NS
Waist circumference (cm)	78.0 ± 11.5	72.8 ± 9.4	< 0.001
Waist circumference z-score	0.32 ± 0.73	0.15 ± 0.71	0.002
HDL-cholesterol (mg/dL)	50.5 ± 14.4	55.0 ± 14.3	< 0.001
HDL-phospholipid (mg/dL)	122.8 ± 24.7	128.5 ± 25.1	0.005
Insulin (μUI/mL)	9.0 ± 7.3	8.7 ± 5.2	NS
Leptin (ng/mL)	6.4 ± 8.3	16.6 ± 10.3	< 0.001
Adiponectin (μg/mL)	11.0 ± 6.6	15.2 ± 8.0	< 0.001
hs-CRP (mg/L)	0.78 ± 1.20	0.64 ± 1.10	NS

*NS*: non-significant.

The average age of children in our study was 14.4 ± 1.2 years, with no significant difference between sexes. No differences in BMI and BMI z-score were observed between males and females, but higher prevalence of overweight (35% versus 25%) and higher values of waist circumference and waist circumference z-score were observed in males than in females. HDL-cholesterol, HDL-phospholipid, leptin and adiponectin concentrations were significantly higher in females than in males. No statistical differences in insulin or hs-CRP concentrations were observed.

### Relationship of hs-CRP with HDL-cholesterol, HDL-phospholipid, insulin, leptin and adiponectin

Due to the non-parametric distribution of hs-CRP to analyze their association with hs-CRP levels, we compare HDL-cholesterol, HDL-phospholipid, insulin, leptin, and adiponectin levels by hs-CRP tertile depending on sex (Table 2). A significant decrease in HDL-cholesterol and HDL-phospholipid levels across hs-CRP tertile was observed in males, but no significant differences were observed in females. A significant progressive increase in leptin levels across hs-CRP tertiles was observed in both sexes. No significant association has been found of insulin or adiponectin with hs-CRP levels.

**Table 2:** HDL-cholesterol, HDL-phospholipid, insulin, leptin, and adiponectin concentrations (mean ± SD) by hs-CRP tertile and sex.

	Males (n = 350)			*p-*value	Females (n = 401)			*p-*value
	1 ≤ 0.17 mg/L	2 0.18–0.63	3 ≥ 0.64 mg/L		1 ≤ 0.15 mg/L	2 0.16–0.46	3 ≥ 0.47 mg/L	
HDL-C (mg/dL)	53.7 ± 14.7	50.8 ± 15.9	46.8 ± 11.6	0.001	55.8 ± 13.6	55.7 ± 14.3	53.5 ± 15.0	0.347
HDL-PL (mg/dL)	127.4 ± 22.5	127.7 ± 28.8	114.7 ± 21.0	0.000	128.2 ± 24.3	132.6 ± 25.1	125.3 ± 25.4	0.115
Insulin (μUI/mL)	8.0 ± 4.0	10.2 ± 11.0	8.8 ± 4.9	0.056	8.8 ± 6.4	8.2 ± 4.3	8.9 ± 4.8	0.486
Leptin (ng/mL)	3.3 ± 4.6	6.1 ± 7.9	10.1 ± 10.0	0.000	14.2 ± 7.9	16.5 ± 10.0	19.2 ± 12.1	0.000
Adiponectin (μg/mL)	11.6 ± 6.9	11.0 ± 6.6	10.4 ± 6.3	0.426	15.6 ± 8.6	15.6 ± 7.8	14.34 ± 7.1	0.348

Spearman correlation analysis (Table 3) showed that HDL cholesterol and HDL phospholipid concentrations were significantly positively correlated with adiponectin levels in both sexes, showing a stronger correlation in females, but were negatively correlated with leptin levels only in males. hs-CRP levels were inversely correlated with HDL cholesterol and HDL phospholipid in males but not in females.

**Table 3:** Spearman’s correlation between hs-CRP, leptin, adiponectin, HDL cholesterol and HDL phospholipid levels by sex.

	Leptin	Adiponectin	HDL-C	HDL-PL
**Males**				
hs-CRP	0.363**	− 0.066	− 0.198**	− 0.235**
Leptin		− 0.010	− 0.160*	− 0.168*
Adiponectin			0.205**	0.269**
HDL-C				0.832**
**Females**				
hs-CRP	0.163*	− 0.069	− 0.052	− 0.053
Leptin		− 0.108	0.020	0.040
Adiponectin			0.346**	0.298**
HDL-C				0.850**

*p < 0.01; **p < 0.001.

Linear regression analyses were used to further evaluate the association of hs-CRP with HDL-cholesterol, HDL-phospholipid, adiponectin and leptin by sex. We observed that HDL-phospholipid and leptin concentrations appeared significantly related to hs-CRP levels in males, in a model accounting for 14.3% of the variance in hs-CRP (Table 4). However, in females, leptin levels appeared as the only significant predictor of hs-CRP, accounting for 7.6% of the variation of hs-CRP concentrations.

**Table 4:** Linear regression models for hs-CRP concentration by sex.

	B ± SE	
	Males	Females
HDL-cholesterol	0.013 ± 0.009	0.003 ± 0.008
HDL-phospholipid	− 0.015 ± 0.005*	− 0.007 ± 0.004
Adiponectin	− 0.015 ± 0.010	− 0.012 ± 0.007
Leptin	0.039 ± 0.008**	0.019 ± 0.005**
R^2^ (%)	14.3	7.6

*p < 0.01; **p < 0.001.

### HDL-cholesterol and HDL-phospholipid levels by hs-CRP tertile after adjusting for adipokines

**Figure 1:**
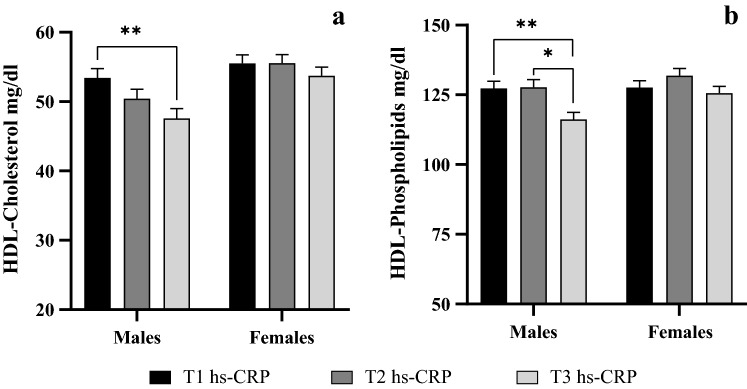
HDL-cholesterol (**a**) and HDL-phospholipid (**b**) levels across hs-CRP tertiles adjusting by leptin and adiponectin. The results are expressed as means and standard error. Data from ANCOVA with Bonferroni’s correction. *p < 0.05; ** p < 0.01. *hs-CRP* high sensitivity C-reactive protein.

To analyze the independent association between hs-CRP levels and HDL-cholesterol and HDL-phospholipid concentrations irrespective of adipokines, we compared HDL-cholesterol and HDL-phospholipid levels by hs-CRP concentration tertiles by sex adjusting for leptin and adiponectin (Fig. 1).

Categorization of hs-CRP by tertiles was: tertile 1 (hs-CRP ≤ 0.17 mg/L), tertile 2 (0.18 mg/L < hs-CRP ≤ 0.63 mg/L) and tertile 3 (hs-CRP > 0.63 mg/L) in males and tertile 1 (hs-CRP ≤ 0.15 mg/L), tertile 2 (0.16 mg/L < hs-CRP ≤ 0.46 mg/L) and tertile 3 (hs-CRP ≥ 0.47 mg/L) in females. In males, after adjusting for leptin and adiponectin, significantly lower mean HDL-cholesterol and HDL-phospholipid concentrations were observed in children in the highest hs-CRP tertile as compared with mean concentrations in children in tertile 1 and tertile 2 of hs-CRP concentration. No significant differences in HDL-cholesterol and HDL-phospholipid concentrations by hs-CRP tertile were observed in females after adjusting by adipokines.

## Discussion

In our study we have analyzed the relationship of hs-CRP levels with HDL-cholesterol and HDL-phospholipid concentrations in a representative population-based sample of adolescents in order to clarify at this age the association between low-grade inflammation, evaluated by hs-CRP, and HDL metabolism. Our results showed that, after adjusting for leptin and adiponectin, hs-CRP concentrations were negatively associated with HDL-cholesterol and HDL-phospholipid concentrations in 12- to 16-year-old males. CRP levels have been associated with low HDL-cholesterol levels in other studies in children^8,9,26–29^ including our study analyzing prepubertal and pubertal children^16^, however, to our knowledge, the negative correlation between hs-CRP and HDL-phospholipid found in our study has not been described previously in children.

Our study design does not provide insight as to whether HDL-cholesterol and HDL-phospholipid concentrations improve the inflammatory state as reflected in lower CRP levels, or whether higher CRP concentrations decrease HDL-cholesterol and HDL-phospholipid. Nonetheless, it seems that HDL acts as an anti-atherosclerotic factor by means of improving inflammatory status reflected in CRP levels. The role of HDL neutralizing the CRP-mediated proinflammatory activity has been demonstrated^30^. The authors suggest a specific role of phospholipid in this protective capacity of HDL against the proinflammatory action of CRP as they showed that the CRP-induced upregulation of inflammatory adhesion molecules was prevented by HDL via their phospholipid components^30^. Additional evidences of the role of phospholipid as anti-inflammatory compounds have been reported^31^. HDL-phospholipids have been associated with acute coronary syndrome and potentially related to plaque rupture or erosion^32^, but its role as a marker of cardiovascular risk remains under study. Reduced HDL-phospholipid content has been associated with development of coronary artery disease in people with very high HDL-cholesterol^33^. Our data add on the knowledge regarding the relationship of HDL-phospholipid with cardiovascular risk, as we describe an association of HDL-phospholipid with inflammatory markers already early in life.

Adiponectin is considered a cardio-protective cytokine due to its anti-inflammatory characteristics^18^ and it could be thought that the association of adiponectin with HDL-phospholipid could help to explain its anti-inflammatory properties. In this cohort, we had already described that high levels of adiponectin were related to higher HDL-cholesterol and HDL-phospholipid concentrations after adjusting by BMI mainly in females^22^. Other studies in adolescents have suggested an inverse association between adiponectin and atherogenic lipoproteins, including small HDL particles, independent of BMI and insulin resistance^34^. However, in this study, we reported no association between hs-CRP concentrations and adiponectin levels. CRP and adiponectin have opposite properties against HDL. The dysregulated elevation of CRP and the reduction of adiponectin may both affect HDL metabolism. According to our findings, we may assume an independent association of adiponectin and CRP with HDL metabolism.

Leptin has also been considered to be acting as a link between inflammation and metabolism in obesity-related disorders^35^ regulating proinflammatory immune responses^36^. In our population we have observed a significant positive association between hs-CRP and leptin levels^16^, which have been observed in studies in adults^25^. However, a significant association between leptin and HDL-cholesterol and HDL-phospholipid levels was observed in males in our cohort, but was not observed in females. Studies in adults also failed to find any association between blood leptin concentration and lipid metabolism^37^. The fact that leptin appears as a predictor of hs-CRP concentrations in regression analysis also suggests an HDL-independent contribution of leptin to hs-CRP levels in adolescents.

An important observation of our study is that the association between hs-CRP with HDL-cholesterol and HDL-phospholipid levels is observed in 12–16-year-old males but not in 12–16-year-old females. We observed an association between hs-CRP and HDL-cholesterol in both sexes in a previous study analyzing younger children^16^. Other studies have described different correlation between hs-CRP levels and HDL cholesterol in adults than in children^38^. All these evidences suggest an age and sex-related factor influencing on the association between CRP and HDL metabolism. A different fat abdominal content could be among the factors that contribute to explain these differences by sex, as we observed a higher waist circumference in males than in females in our population. However, further studies are needed to clarify the reasons behind differences by sex.

Our study has some limitations that we should mention. First, the detection lower limit for the measurement of hs-CRP concentration is set at 0.15 mg/L, which makes difficult statistical analysis. Second, the lack of information regarding the Tanner stage, preventing us from using this datum as a covariate in our analysis, as well as the fact that the diagnosis of delayed or precocious puberty may not be completely accurate as it was informed by the parents. Nevertheless, our study has the strength of including a large sample size from a homogeneous population representative of children population of this age.

In conclusion, in our study we report an inverse association of CRP concentrations with HDL-cholesterol and HDL-phospholipid levels in 12- to 16-year-old males, independently of adipokine levels. This association is not evident in 12- to 16-year-old females in whom hs-CRP concentrations seem to be related to leptin levels. To our knowledge, our study is the first to show a significant negative correlation between CRP and HDL-phospholipid in adolescents. Our data suggest the protective role of HDL-cholesterol and HDL-phospholipid concentrations in relationship with low grade inflammation in males at this age.

## Methods

### Subjects

Our population-based sample comprises 751 adolescents aged 12 to 16 years without any chronic disease. Subjects were participants of a cross-sectional study examining cardiovascular risk factors in Spanish schoolchildren^22^ of whom we have information regarding plasma concentrations of lipid parameters, including HDL-cholesterol and HDL-phospholipid, insulin, leptin, adiponectin and hs-CRP levels. Unfortunately, due to the design of our study, studying children at schools, no information on Tanner stage is available in our study, but information on age at menarche was available in order to classify the pubertal status of the girls. All children reported by parents to be suffering from chronic disease, including precocious and delayed puberty, were excluded from the study. Parents or legal guardians were required to provide written informed consent for their children to participate in the study. The study protocol was approved by the Ethics Committee of Clinical Investigation of the Instituto de Investigación Sanitaria-Fundación Jiménez Díaz (protocol code: PIC016-2019 FJD). The investigation fulfils the principles contained in the Declaration of Helsinki and subsequent reviews, as well as the prevailing Spanish legislation on clinical research in human subjects.

A team consisting of one physician and several nurses was in charge of blood extractions and physical measurements.

### Anthropometric data

Measurements were taken with the adolescents lightly dressed and barefoot. Height was measured to the millimeter using a portable stadiometer, and weight was recorded to the nearest 0.1 kg using a standardized electronic digital scale. From these measurements, BMI (weight in kilograms/height in meters squared) was computed. Waist circumference (WC) was measured at the narrowest point between the lowest rib and the uppermost lateral border of the right iliac crest, as described^22^. BMI-z score and WC-z score were calculated according to predetermined values in a reference population.

### Biochemical data

Blood samples were collected in the morning after overnight fasting by venipuncture into Vacutainer tubes. Samples were kept on ice and sent to the laboratory for analysis. Once centrifuged, fractions were separated and frozen at − 70 °C. As described^22^, cholesterol and phospho-lipids were measured enzymatically (Menarini Diagnostics, Florence, Italy) with an RA-1000 Autoanalyzer (Technicon Ltd, Dublin, Ireland). HDL-cholesterol and HDL-phospholipids were measured after precipitation of apo B-containing lipoproteins with phosphotungstic acid and Mg (Roche Diagnostics, Madrid, Spain). Insulin concentrations were measured using a commercial kit (BI-Insulin IRMA, Bio-Rad, France). Adiponectin and leptin levels were determined by ELISA using commercially available kits according to the manufacturer’s protocols (Human Adiponectin E-09, Mediagnost Reut-lingen, Germany; Leptin EIA-2395, DRG, Germany). CRP concentrations were measured using a high sensitivity C-Reactive Protein ELISA commercial kit (hs-CRP SK00080-02, Aviscera Bioscience, Inc., Santa Clara, USA). Hs-CRP levels were measured with a detection limit of 0.15 mg/L.

### Data analysis

Statistical analysis was performed using the IBM SPSS software package (Chicago, Illinois, Version 21.0) and GraphPad Prism statistical software (San Diego, California, Version 8). Children with hs-CRP levels greater than or equal to 10 mg/L were excluded from the study to avoid the influence of acute infection. The Kolmogorov–Smirnov test was used to analyze whether the variables under study were normally distributed. Variables that were not normal distributed were log transformed prior to analysis. Student’s t-test was used to analyze differences by sex in the variables under study. An ANOVA test was used to compare means of biochemical variables (HDL components (phospholipid, cholesterol), leptin, adiponectin, insulin) by hs-CRP tertiles. Linear regression models were used to analyze the independent relationship of the variables under study with hs-CRP concentrations by sex. To further analyze the relationship between hs-CRP and HDL-cholesterol and HDL-phospholipid levels, analyses of covariance (ANCOVA) were used to compare HDL-cholesterol and HDL-phospholipid concentrations by hs-CRP concentration tertile depending on sex after adjusting by the corresponding confounder variables.
